# Teamwork Makes the Dream Work: A Team-Based Approach to Achieving AFU Designation

**DOI:** 10.1093/geroni/igaf122.1003

**Published:** 2025-12-31

**Authors:** Audrey Perry

**Affiliations:** Frontier Nursing University, Versailles, Kentucky, United States

## Abstract

The university created an Age-Friendly University (AFU) application team representative of the diverse services and stakeholders that assess, evaluate, support, and create Age-Friendly university and community opportunities. After creating a diverse team, reviewing AFU principles, and establishing goals and team agreements, university staff, faculty, and community partners effectively and swiftly contributed to the AFU application using principles of effective teams. The team submitted a successful application based on past and future Age-Friendly collaborative efforts. Additionally, the team-based, goal-oriented, principle-driven approach clarified future collaboration between the university and the community. Establishing a diverse application team resulted in short-term and sustained systems-level AFU efforts.

